# A Manual of Obstetrics

**Published:** 1889-12

**Authors:** 


**A Manual of Obstetrics.** By A. F. A. KING, A. M., M. D., Professor of Obstetrics and Diseases of Women and Children, in the Medical Department of the Columbian University, Washington, D. C., and in the University of Vermont, etc., with 141 illustrations. Fourth edition. Philadelphia: Lea Brothers & Co. 1889.

The fourth edition of this valuable little work on Obstetrics, from the pen of Professor King, following so soon after the previous edition, shows the appreciation of the profession and of medical students for a condensed and concise treatise on the rudiments and essentials of obstetric science. The busy practitioner and the overworked student have not the leisure to wade through the more elaborate works, and they find in this compendium all that is essential for their needs. The present edition presents an increase in the number of pages and of illustrations. Two new chapters have been added on the subjects of intercurrent diseases of pregnancy and the resuscitation of still-born children. In other respects, also the work contains the latest improvements and advances in this special department of medicine.

We have taken occasion to recommend the work to our students because of its brief, concise and accurate presentation of the principles of the science, and also for the reason of its economy in cost, the latter being an important matter with many medical students.

